# Supplementary figures and images for: Genetic diversity of Microsporidia in the circulatory system of endemic amphipods from different locations and depths of ancient Lake Baikal
Source: PeerJ. 2018 Aug 2;6:e5329. doi: 10.7717/peerj.5329 (PMC6076988; doi:10.7717/peerj.5329)

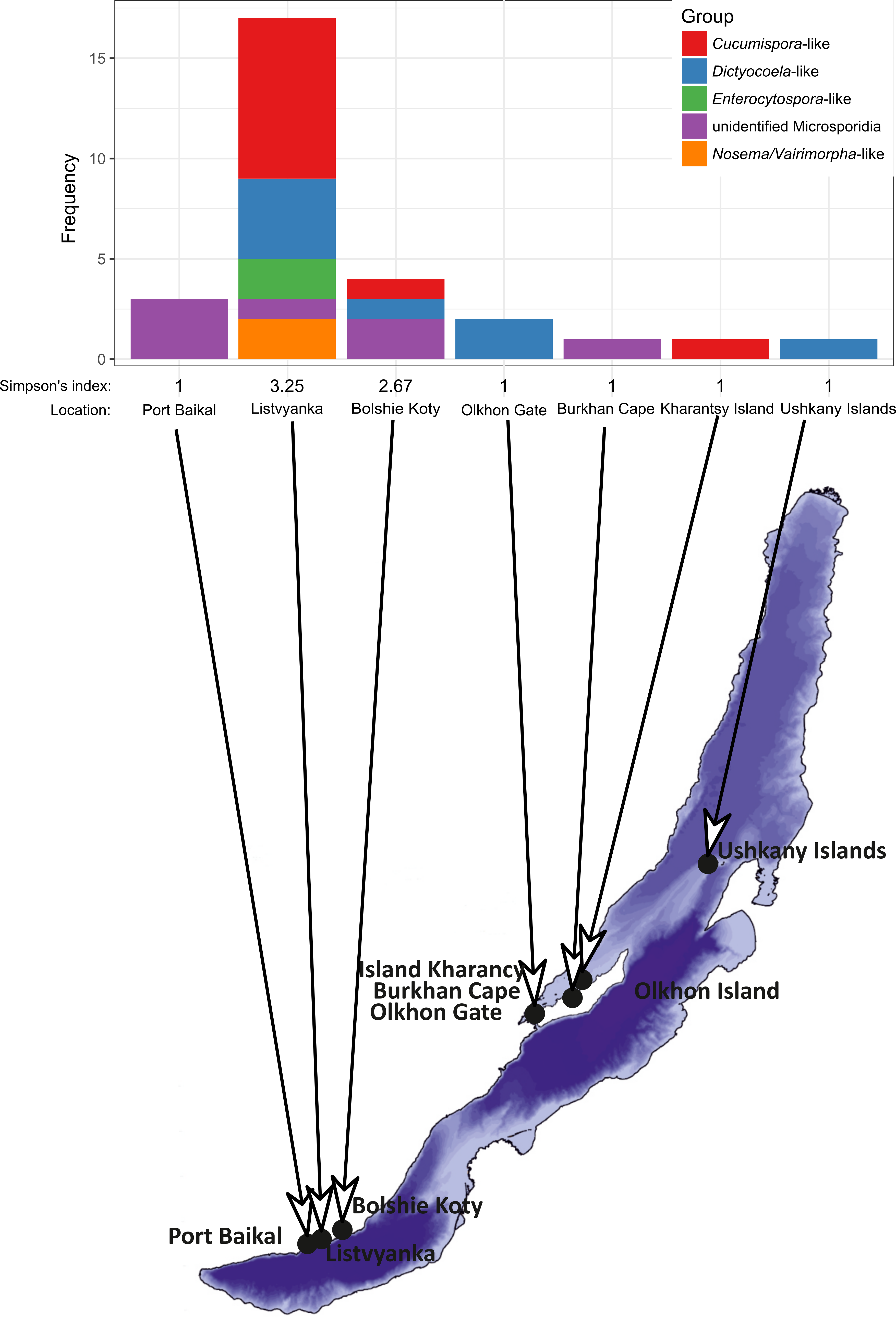

Supplement: Figure S1 — The vertical axis features the number of microsporidian isolates. [file peerj-06-5329-s003.png]
